# Effects of electronic coupling and electrostatic potential on charge transport in carbon-based molecular electronic junctions

**DOI:** 10.3762/bjnano.7.4

**Published:** 2016-01-11

**Authors:** Richard L McCreery

**Affiliations:** 1 Department of Chemistry, University of Alberta, Edmonton, AB, Canada and National Institute for Nanotechnology, National Research Council, Canada

**Keywords:** molecular junction electron transport density functional theory molecular devices

## Abstract

Molecular junctions consisting of 2–20 nm thick layers of organic oligomers oriented between a conducting carbon substrate and a carbon/gold top contact have proven to be reproducible and reliable, and will soon enter commercial production in audio processing circuits. The covalent, conjugated bond between one or both sp^2^-hybridized carbon contacts and an aromatic molecular layer is distinct from the more common metal/molecule or silicon/molecule structures in many reported molecular junctions. Theoretical observations based on density functional theory are presented here, which model carbon-based molecular junctions as single molecules and oligomers between fragments of graphene. Electronic coupling between the molecules and the contacts is demonstrated by the formation of hybrid orbitals in the model structure, which have significant electron density on both the graphene and the molecule. The energies of such hybrid orbitals correlate with tunneling barriers determined experimentally, and electronic coupling between the two graphene fragments in the model correlates with experimentally observed attenuation of transport with molecular layer thickness. Electronic coupling is affected significantly by the dihedral angle between the planes of the graphene and the molecular π-systems, but is absent only when the two planes are orthogonal. Coupling also results in partial charge transfer between the graphene contacts and the molecular layer, which results in a shift in electrostatic potential which affects the observed tunneling barrier. Although the degree of partial charge transfer is difficult to calculate accurately, it does provide a basis for the “vacuum level shift” observed in many experiments, including transport and ultraviolet photoelectron spectroscopy of molecular layers on conductors.

## Introduction

The field of Molecular Electronics investigates the behavior of molecules as elements in electronic circuits, with the intent of exploiting variations of molecular structure to realize unusual electronic functions [1–4]. Charge transport through single molecules and through ensembles of molecules in nanoscale (1–20 nm) films has been studied experimentally since the late 1990s, with particular interest in how structure affects transport. The experimental paradigms are numerous [1,5], but they all share a common phenomenon of electrical communication between molecules and “contacts” made from conventional conductors or semiconductors. An extensive theoretical effort has accompanied the experiments [6–12], with one objective being a rational design of particular molecular structures to realize desirable electronic responses. The driving force for the field is the possibility of electronic components that are smaller than existing transistors or diodes, have unusual properties not possible with conventional semiconductors, use less power, or are cheaper than existing microelectronics. A basic element of Molecular Electronics is the “molecular junction (MJ)” consisting of molecules oriented between two conductors, with charge transport through the molecular layer. The vast majority of existing junction structures are based on metal/molecule bonding such as the Au/thiol self-assembled monolayers [13–21], Langmuir–Blodgett films on metals [22–23], or molecules bonded to silicon [24–27].

Our research group has developed a distinct approach based on conducting carbon substrates with covalently bonded molecular layers applied by reduction of diazonium reagents [2,28–33]. The strong carbon–carbon bonds result in thermally stable MJs (−260 to +350 °C), which operate for billions of continuous current–voltage cycles over a period of several months, and have a shelf life of at least seven years in air. The sp^2^ carbon substrate and aromatic molecular layer introduce a special property into carbon-based MJs, in that they contain a covalent, conjugated “contact” between two aromatic π-systems. The reproducibility, reliability, and operating life of carbon-based MJs resulted in an application in audio processing of electronic music, available commercially in 2015 as an accessory for electric guitars. The fundamental structural difference between metal/molecule and carbon/molecule MJs is expected to result in possibly significant differences in electronic behavior. In particular, the electronic interactions between the molecules and the contacts might be quite different, leading to changes in transport barriers and junction conductance [2,34–35]. A consequence of electrode/molecule interactions is that “vacuum level shifts” can change the transport barriers significantly from those based on the free molecule energy levels [14,31,36–40]. An unexpected result likely due to this effect is the “compression” of tunneling barriers predicted to range over 2.4 eV based on the free molecule energy levels to an observed range of 1.3 ± 0.2 eV in carbon-based MJs [31,34]. The current report describes the application of density functional theory (DFT) to carbon-based MJs, in order to investigate which aspects of junction behavior are attributable to the unique carbon–carbon bonding at one or both contacts of the molecular junction.

A simple model based on single molecules and oligomers bonded to small graphene fragments representing the sp^2^ carbon contacts provides insights into how electronic interactions between the molecules and the contacts affect tunneling barriers and local electrostatic potential. The approach is an extension of a detailed theoretical analysis of graphene/molecule interfaces [34], with the addition of a second sp^2^ carbon conducting contact. Although real carbon-based MJs are structurally complex, the simple model yields significant correlations between theoretical predictions and experimental observations. In particular, four general questions are considered to evaluate the model: First, how does bonding between the carbon contact and the molecular layer alter the orbital energies and electron distributions? Second, does the calculated electronic coupling across the carbon MJ correlate with the observed junction conductance? Third, how does charge transfer between the graphene contacts and molecular layers affect the transport barriers? Fourth, can the model predict the behavior of carbon MJs to provide guidance for molecule synthesis and junction fabrication? Throughout the discussion, the main purpose is identification of the major factors affecting the electronic behavior of the completed carbon/molecule/carbon MJ, in addition to quantitative correlations with experimental results where possible.

## Experimental

Common DFT procedures were used, in part to maximize availability to potential users. Gaussian09 version 9.5 (revision D.01 Windows 64 bit) and Gaussview 5.0.9 software packages were used for all calculations and visualization of molecular structures and orbitals, using the B3LYP functionals and 6-31G(d) basis set unless stated otherwise. Although we have reported more sophisticated treatments with significant computational demands [34], the calculations reported here were carried out with a Pentium 4-processor desktop computer. The B3LYP functionals were used in the current report rather than the BLYP functionals used previously, partly because B3LYP is generally more accurate, but also because it was not available for the more complex models in the previous work. In most cases the maximum computing time was a few hours using the multiprocessor version of Gaussian09 for Windows. Orbital visualization with Gaussview used the default isovalue of 0.02, which is commonly used to represent the majority of the electron density. Predictions of charge transfer within model molecules used the Mulliken charges calculated during the DFT analysis. There is some uncertainty about the most accurate calculations of local charges [41], but the trends are consistent with electronegativity and Hammet parameters, and are useful for estimating electrostatic effects on barriers, as described below.

### The model

**Figure 1:**
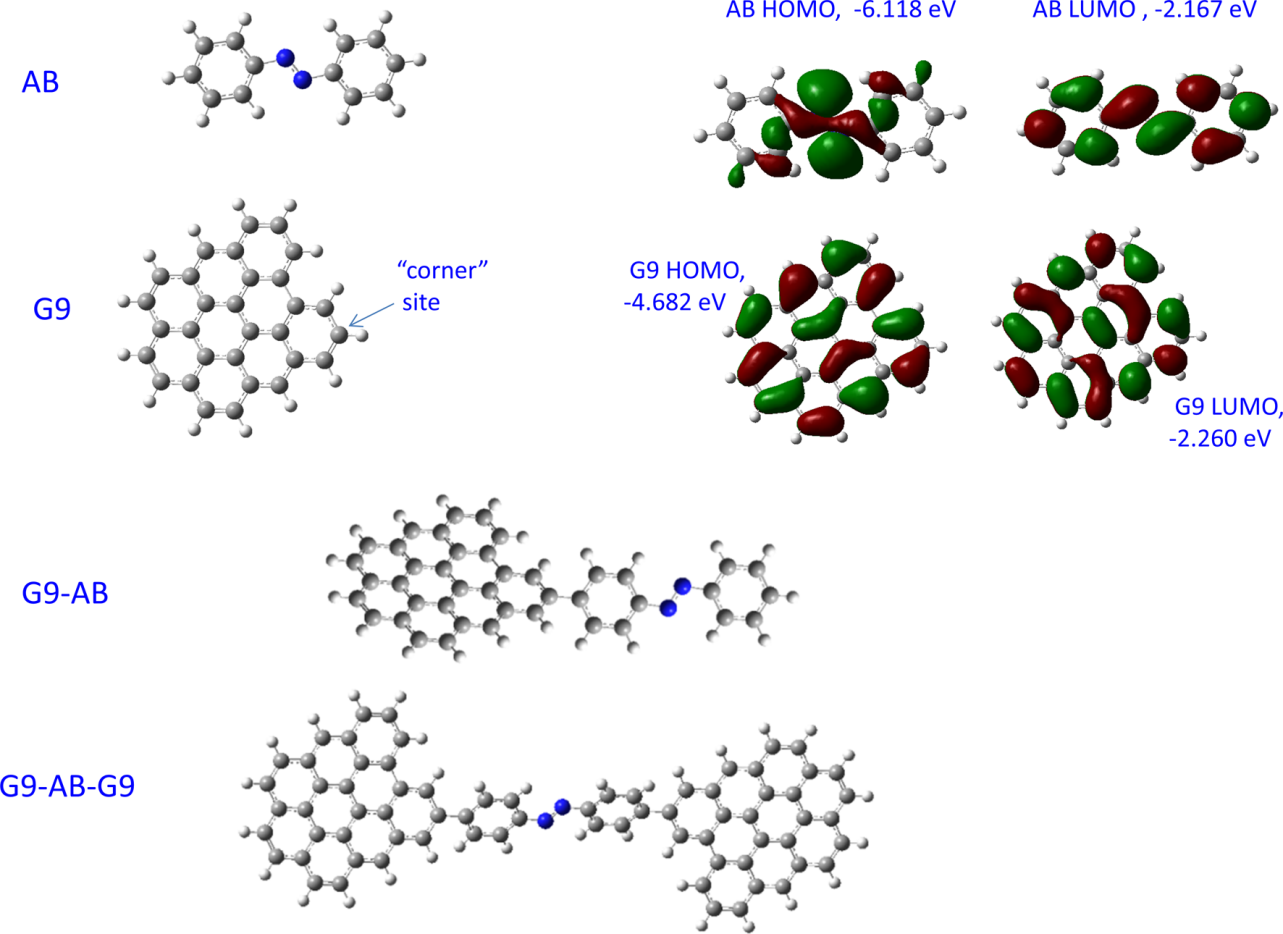
Structures of azobenzene (AB), 9-ring graphene fragment (G9), AB bonded to a G9 “corner” site (G9–AB), and AB bonded to two G9 rings to represent a carbon-based molecular junction (G9–AB–G9). Also shown are the HOMO and LUMO orbitals of isolated AB and G9, as indicated.

The molecules subjected to DFT analysis are shown in Figure 1, for the case of an azobenzene (AB) molecule covalently bonded to an edge site in a 9-ring graphene fragment, denoted “G9”. We described the rationale for investigating edge-bonding previously [2,34,42–43], and it is the most likely site in real devices. Since the carbon surface is disordered and the molecular layers are often multilayers, the real system will have a range of dihedral angles between the molecular aromatic rings and those of the graphene. The important effect of this dihedral angle will be discussed below. The choice of a “corner” site rather than the more common “armchair” and “zig-zag” sites reduces steric hindrance at the molecule–G9 bonding site. In a detailed discussion of the bonding site with higher level theory, we showed that the site type had a much smaller effect on tunneling barriers than the dihedral angle [34]. The corner site shown in Figure 1c and Figure 1d will be used in all calculations and figures unless noted otherwise.

Also shown in Figure 1 are the highest occupied molecular orbitals (HOMO) and lowest unoccupied molecular orbitals (LUMO) for G9 and AB. In all cases, the orbital energies are stated relative to a vacuum reference, consistent with commonly used conventions, including that of Gaussian09 software. Whenever the parameters for a planar molecular configuration are calculated, the molecule was first optimized, then the dihedral angles between the π-system of the molecule and the G9 plane were set to zero and the orbital energies recalculated. The discussion starts with consideration of the effects of covalent bonding between the graphene “contact” and the aromatic molecule in the molecular layer.

## Results and Discussion

### 1 Orbital energies and electron distribution

**Figure 2:**
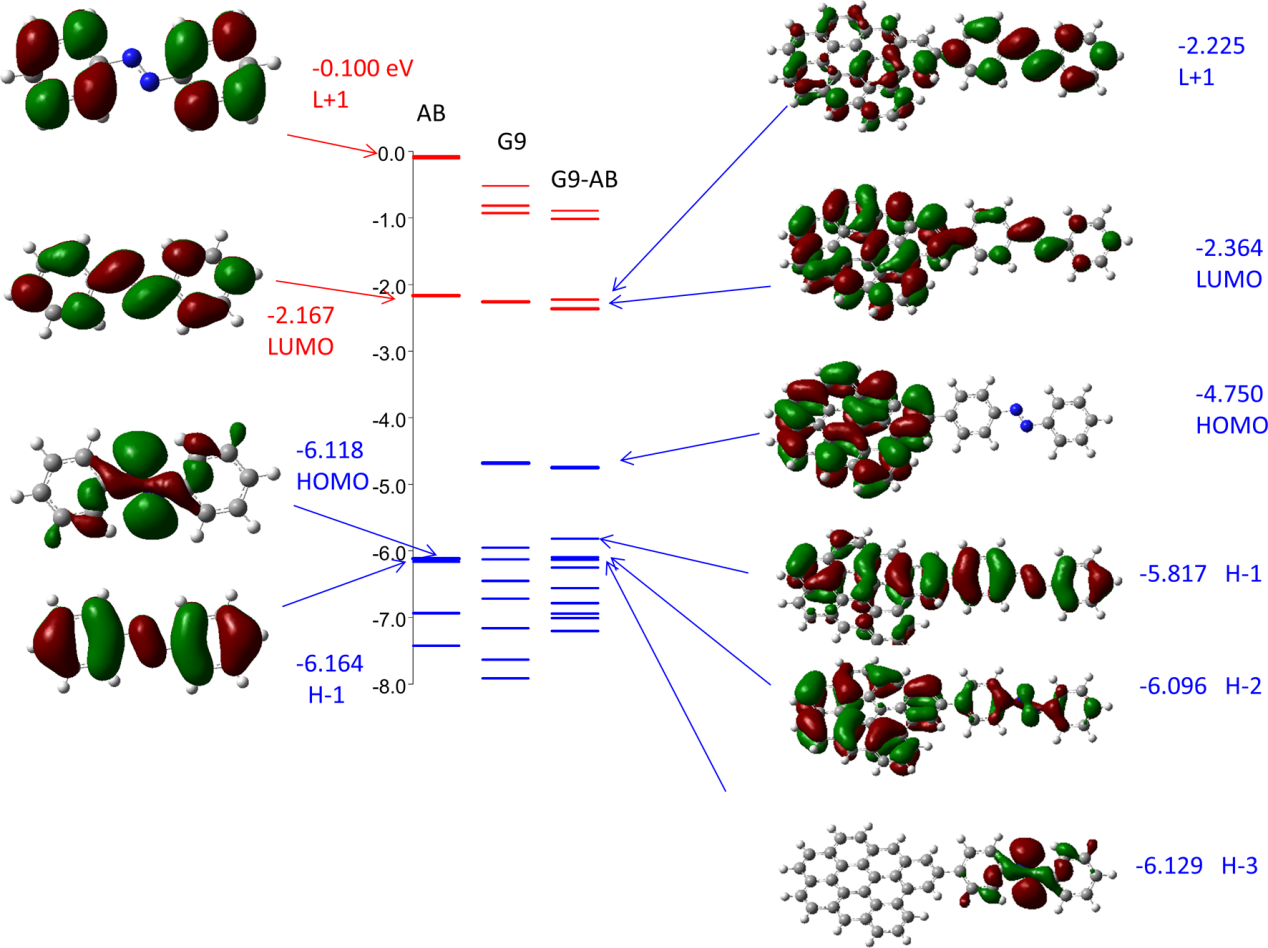
Energy level diagram comparing orbital energies for isolated AB and G9, compared to the G9–AB cluster (middle), all in eV relative to a vacuum reference. Selected orbitals and their energies (in eV) are surround the diagram. Molecules are in their optimized geometries, with a 37° dihedral between G9 and AB planes in G9–AB.

Given that both the molecular layer and the graphitic contact are aromatic π-systems, there likely are significant electronic interactions between the two, which should alter the electron distribution and energies compared to the separated molecule and contact. Figure 2 shows the energies of orbitals near the HOMO and LUMO for G9, AB, and the G9–AB combination.

The electron density distributions for selected orbitals are also shown, all for the case of the optimized geometry, which has a dihedral angle between the G9 plane and the AB phenyl ring of 37°. The G9 orbitals are not shown, but they all have the general appearance of the HOMO and LUMO shown in Figure 1, with extensive delocalization over the nine benzene rings. The orbitals of the G9–AB combination in the right side of Figure 2 provide clear indications that there are significant interactions between the orbitals of G9 and AB molecules upon covalent bond formation. We will refer to orbitals of G9–AB (and later G9–AB–G9) as “system orbitals”, since they often differ significantly from the orbitals of the free AB and G9 molecules. The system L+1, LUMO, H−1 and H−2 orbitals all show shared electron density across both G9 and AB, while the system HOMO at −4.75 eV is localized on G9 and has an energy close to that of the free G9 HOMO (−4.75 eV). The system H−3 orbital resembles the original AB HOMO, and is localized on the AB molecule in the G9−AB system. It is important to note here that the system H−1 and H−2 orbitals are “hybrid” orbitals formed when the AB is bonded to the G9 cluster. G9–AB is a different molecule from the individual components, and permits delocalization not possible in the free molecules. The system HOMO and H−3 could be considered remnants of the corresponding orbitals in the free molecules, but H−1 and H−2 are characteristic only of the G9–AB system. The importance of these orbitals to transport in an AB molecular junction will be considered below.

**Figure 3:**
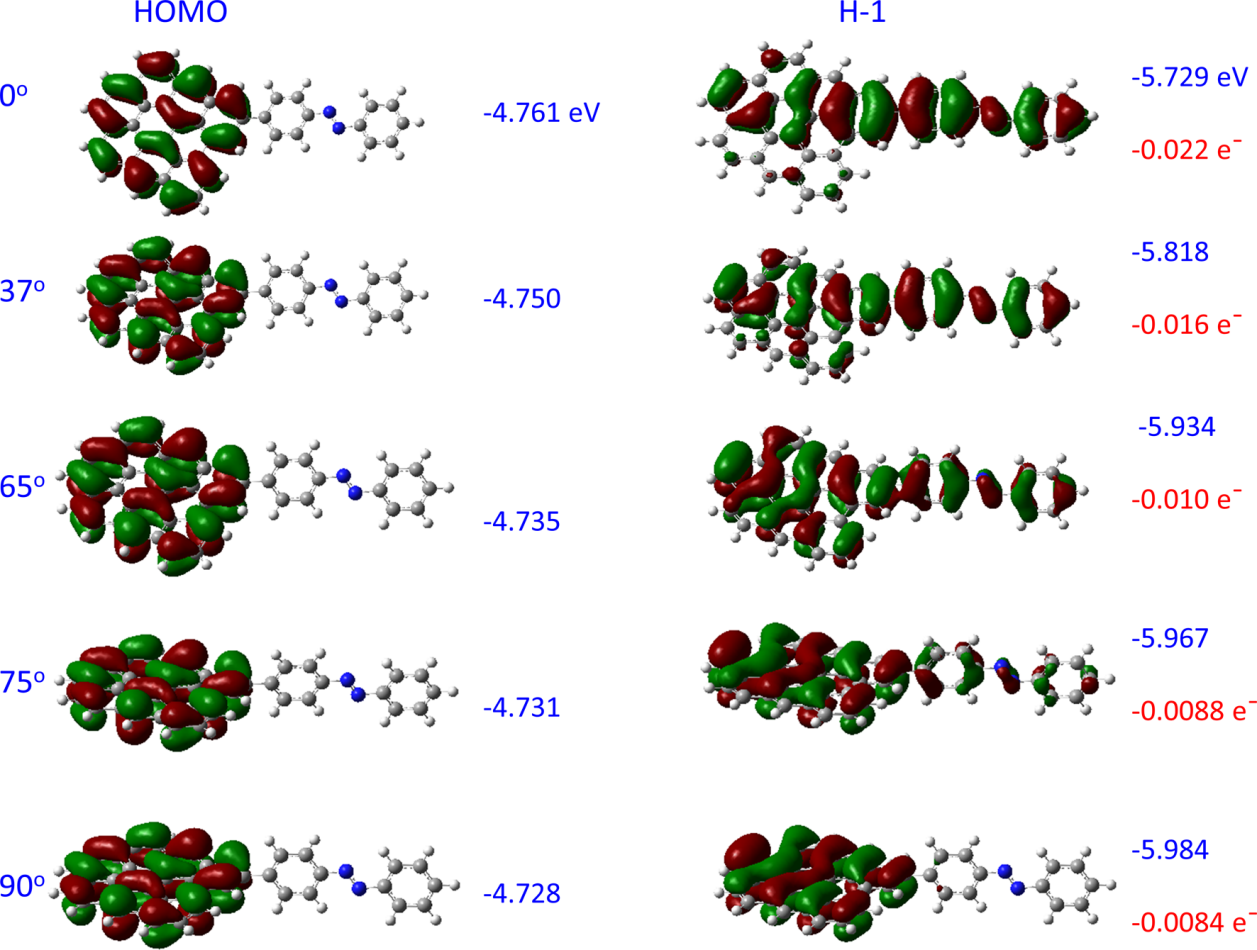
HOMO and H−1 orbitals for G9–AB for a range of dihedral angles between the G9 plane and AB aromatic rings, indicated at left. Orbital energies for all orbitals are indicated in blue, while the sum of the Mulliken charges on the AB moiety is indicated in red. A negative Mulliken charge indicates partial electron transfer from G9 to AB upon forming the covalent G9–AB cluster.

Since the G9 aromatic systems and the AB phenyl rings are conjugated and covalently bonded, we expect that the degree of interaction between the two π-systems should depend on the dihedral angle between the G9 plane and the AB rings. Figure 3 shows the HOMO and H−1 orbitals for G9–AB with five dihedral angles, along with their energies. Additional orbital energies are listed in Table 1. As expected, the degree of delocalization varies significantly with dihedral angle, becoming larger as the dihedral angle approaches zero. Two additional observations are important to subsequent discussions about transport and energy barriers. First, the HOMO is localized nearly completely on the G9 fragment, and is largely unaffected by the dihedral angle, with its energy varying by 33 meV between 0 and 90° dihedrals. In contrast, the H−1 orbital is delocalized over both G9 and AB fragments, and has a much larger dependence on dihedral, with a range of 256 meV. Second, the sum of the Mulliken charges on the AB moiety of G9–AB is negative, indicating partial charge transfer between the G9 and AB portions of the G9–AB system. The extent of charge transfer varies with angle, from 0.8% of an electron to 2.2% as the dihedral angle is decreased from 90 to 0°.

**Table 1:** Orbital energies for G9, azobenzene (AB) and G9–AB.

molecule	dihedral angle^a^, °	stability, eV^b^	LUMO, eV	HOMO, eV	H−1, eV	H−2, eV	charge on AB^c^

free AB			−2.167	−6.118	−6.164	−6.935	

free G9			−2.260	−4.682	−5.950	−6.126	

G9–AB	0	0.124	−2.401	−4.761	−5.729	−6.102	−0.022
	20	0.050	−2.390	−4.758	−5.757	−6.100	−0.020
	37.3	0.000	−2.364	−4.750	−5.818	−6.096	−0.016
	45	0.010	−2.351	−4.745	−5.849	−6.095	−0.014
	65	0.073	−2.321	−4.735	−5.934	−6.103	−0.010
	75	0.108	−2.317	−4.731	−5.967	−6.117	−0.0088
	90	0.128	−2.309	−4.728	−5.984	−6.146	−0.0084

^a^Between G9 and AB planes; ^b^relative to optimized structure (37.3° dihedral angle); ^c^total Mulliken charge on AB moiety.

This small degree of charge transfer should not be considered “reduction” in the electrochemical sense, but rather a redistribution of charge due to the different electronegativity of portions of the entire molecule. For example, similar calculations for G9 attached to different molecules show charge transfer ranging from 0.0005 e^−^ for G9–biphenyl to 0.049 e^−^ for G9–nitrophenyl. Although the predicted charge transfer is small, it has significant consequences, as discussed below in section 3.

**Figure 4:**
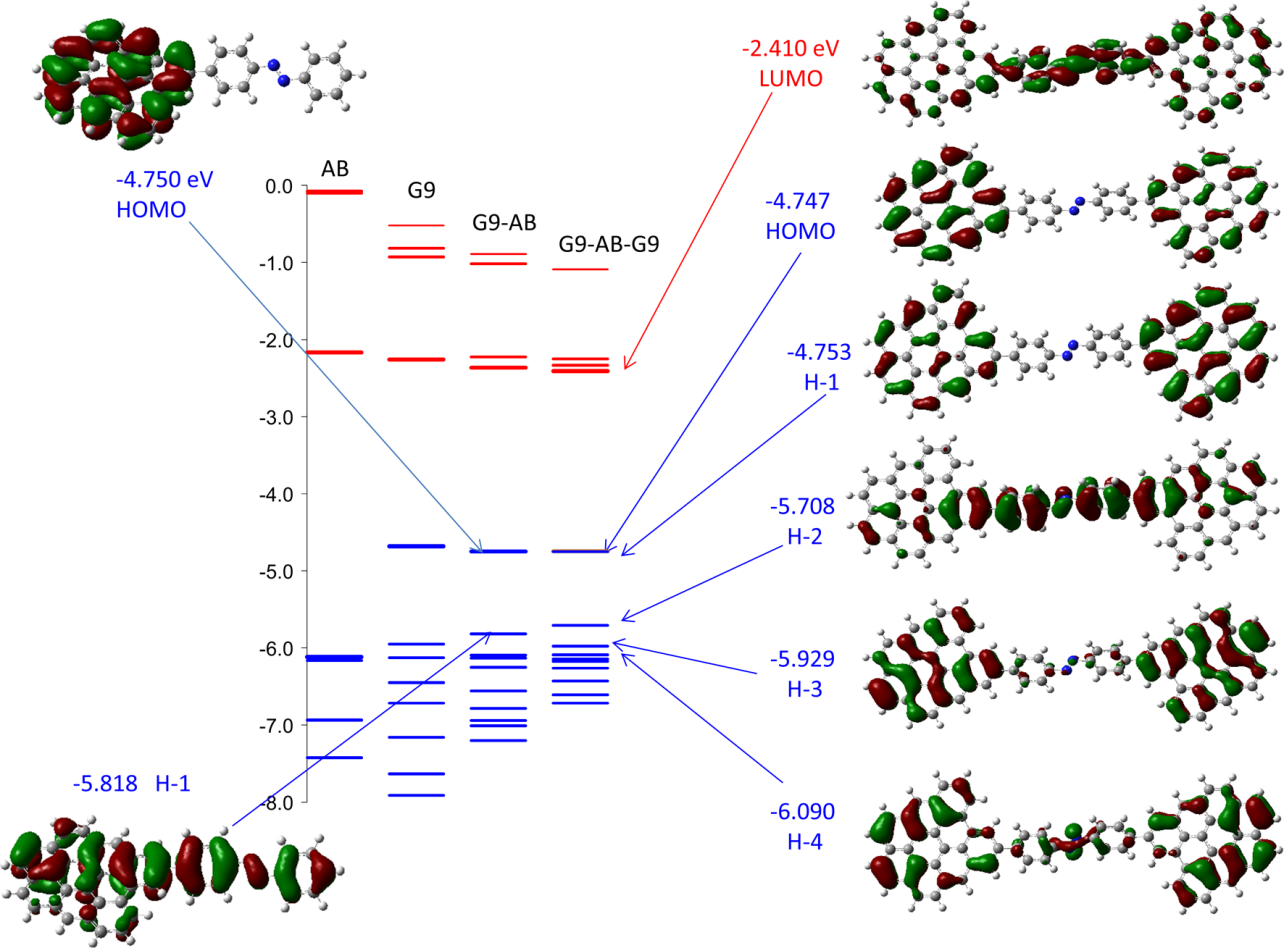
Orbitals for G9–AB–G9 added to the orbital energy diagram of Figure 2. All structures are presented in their optimized geometry, with dihedral angles between AB and G9 of 36 and 39°.

We have reported various top contacts for MJs with carbon substrates, including Cu [31,33,35] ], TiO_2_ [44–46], Si [47], and e-beam deposited carbon (eC) [30,43]. The “all-carbon molecular junction” is of particular interest, since it is more stable than Cu toward voltage and temperature extremes and is less prone to electromigration and oxidation than most metals [43]. The covalent bond between an sp^2^-hybridized carbon substrate and the aromatic molecular layer represented by the G9–AB model structure is well characterized, but the nature of the “contact” between the top eC layer and the molecular layer in all carbon MJs is currently unknown. We showed that the Raman spectrum of the molecular layer is not significantly altered by eC/Au deposition [43], but a covalent bond is likely to form given the reactivity of carbon atoms and clusters generated in an e-beam source. With these caveats in mind, consider the G9–AB–G9 model structure shown in Figure 1, which represents an idealized structure of a single AB molecule covalently bonded to two graphene fragments. The orbital energies for an optimized G9–AB–G9 molecule having 37° dihedral angles between the AB plane and the G9 rings are shown in Figure 4, along with the electron distributions of several orbitals.

**Figure 5:**
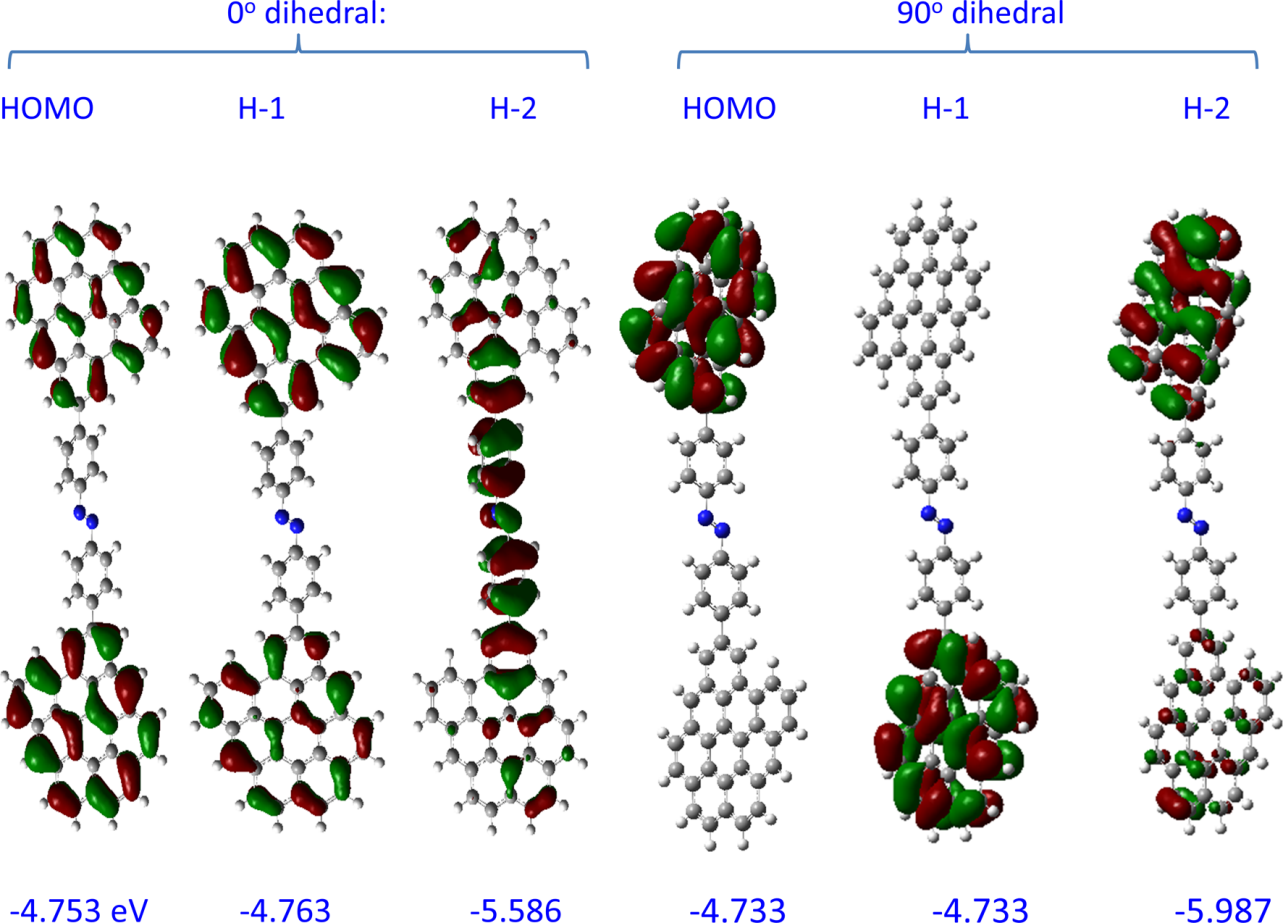
Effect of dihedral angle on orbital electron distributions in G9–AB–G9. In the 0° case, the G9s and AB are coplanar, while in the 90° case the G9s are coplanar but the AB plane is perpendicular to both G9s. Orbital energies are shown below each structure, in eV.

Note that the HOMO and H−1 orbitals of G9–AB–G9 have energies close to that of free G9, and that the electron density is localized on the graphene fragments. The H−2 orbital of G9–AB–G9 is analogous to H−1 for G9–AB, and has electron density that extends across the AB molecule and onto both G9 fragments. Figure 5 shows the effect of dihedral angles in the G9–AB–G9 system, with the HOMO, H−1 and H−2 orbitals shown when both dihedral angles are 0° to make a fully planar geometry, and both are 90°, for comparison to the 36°and 39° angles in the optimized case of Figure 4. Note that the G9 rings are coplanar in all cases, and the AB molecule rotates relative to this common plane as the dihedral angle is increased. Not surprisingly, the delocalization across the entire molecule for H−2 is absent when the AB and G9 rings are orthogonal. Note also that the H−2 energy varies by 400 meV as the dihedral angle increases from 0 to 90°.

### 2 Electronic coupling across the molecular layer

A primary motive for considering the G9–AB and G9–AB–G9 model structures is the ability to predict electronic coupling between the contacts and the molecules. Electronic coupling is a factor in most treatments of electron transfer, including Marcus theory, tunneling between two sites, and transport through numerous sites in the “tight binding” models of conducting solids. In all three cases, stronger electronic coupling increases the electron transfer and/or the conductance relevant to the chemical system considered. These theories will not be reviewed here, but all contain a factor for electronic coupling, often denoted “*t*”, or “*H*_ab_”. For example, the “tight binding” model for off-resonance transport in molecular tunnel junctions predicts that the transport is proportional to *t^2^*^(^*^N ^*^− 1)^, where *N* is the number of “sites” between the contacts [48]. For transport in conducting polymers, *H*_ab_ is related to charge propagation between polarons both along the chain and between different chains [49–50]. These theories include a barrier height as well as a coupling term, both of which affect the overall electron transfer rate. A case relevant to the current discussion is coupling between two orbitals in separate but identical molecules, for example the HOMO orbitals of G9. By definition, these orbitals have identical energies when the two G9 fragments are widely separated. When they are brought into close proximity, electrons in both HOMO orbitals interact to generate “splitting”, creating two hybrid orbitals with energies above and below the HOMO of the free molecules. For example, the free G9 HOMO energy is −4.682 eV, while the HOMO and H−1 orbitals of two parallel G9 planes separated by 1.2 nm have equal energies of −4.682 eV, with no indication of electronic coupling. The parameter “*t*” is given by Equation 1, and is zero in this case.

[1]
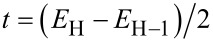


Values of *t* were calculated for two G9 clusters in a vacuum, oriented in parallel or edge to edge, with the latter representing an idealized vacuum gap consisting of a G9–molecule–G9 system with the molecule absent. Several orientations are listed in Table 2, and Figure S1 (Supporting Information File 1) illustrates the different orientations. For the case of parallel, face-to-face G9 planes, the original HOMO levels (H and H−1 in Table 2) change from a common value of −4.650 eV for 1.2 nm separation to −3.740 and −4.982 eV for 0.3 nm separation, clearly indicating an electronic perturbation in the absence of covalent bonding. The value of *t*_H/H−1_ determined from Equation 1 increases from 0 to 621 meV, with significant electronic interactions occurring below 0.6 nm between planes. Also listed in Table 2 are the same energies for the case of edge-to-edge spacing with the G9 rings in the same plane. Electronic coupling is much weaker in this case, and also depends on the dihedral angle between the G9 planes. Note that *t*_H/H−1_ for orthogonal edge-oriented G9s is an order of magnitude lower than for parallel edge oriented planes, and both of these are approximately two orders of magnitude lower than that observed for basal-basal orientation.

**Table 2:** Orbital energies and electronic coupling between G9 molecules.

	gap^a^ between C–C, nm	dihedral angle between G9 planes, °	stability, eV	LUMO, eV	HOMO, eV	H−1, eV	*t*_H/H−1_, meV	*t*_H−2/H−3_, meV	*t*_L/L+1_, meV

free G9				−2.260	−4.682	−5.950			

G9–G9 edge orientation	1.208	0	−0.006	−2.266	−4.688	−4.688	0.0	0.0	0.0
	0.600	0	−0.003	−2.278	−4.700	−4.700	0.0	0.0	0.0
	0.400	0	0.053	−2.290	−4.712	−4.713	0.1	1.1	0.0
	0.350	0	0.333	−2.293	−4.715	−4.715	0.3	3.3	0.0
	0.316	0	1.086	−2.294	−4.716	−4.717	0.5	6.7	0.0
	0.252	0	4.276	−2.297	−4.718	−4.727	4.5	5.7	0.0

G9–G9 edge separated by 0.4 nm	0.400	0	0.053	−2.290	−4.712	−4.713	0.1	1.1	0.0
	0.400	37	0.050	−2.288	−4.709	−4.710	0.1	1.0	0.1
	0.400	60	0.044	−2.285	−4.707	−4.707	0.3	0.5	0.3
	0.400	90	0.042	−2.284	−4.705	−4.706	0.3	0.1	0.3

G9–G9 parallel basal orientation	1.200	0	0.005	−2.228	−4.650	−4.650	0.0	0.0	0.0
	0.600	0	0.038	−2.159	−4.577	−4.581	2.3	43.1	2.4
	0.500	0	0.070	−2.158	−4.528	−4.577	24	25	27
	0.400	0	0.168	−2.251	−4.366	−4.665	150	90	157
	0.300	0	3.910	−2.779	−3.740	−4.982	621	14	679

^a^Distance between nearest C atoms in separate G9 molecules.

Venkataraman, et al. considered the relevance of electronic coupling in Au/molecule/Au single molecule junctions by calculating 4*t^2^* for the frontier Au orbitals in the contacts, as modulated by the intervening molecular “bridge” [8]. Changing the substituents on the diamine “bridge” varied the junction conductance, and the theoretical 4*t^2^* approximately tracked the observed conductance. We use here a conceptually similar approach for carbon-based molecular junctions by considering the electronic coupling between the G9 “contacts” of the G9–molecule–G9 system depicted in Figure 1. We consider the unmodified G9 clusters of Table 2 as models of a vacuum gap between two graphene “contacts”, with either edge-to-edge or basal–basal orientations of the two G9 planes. These will be compared to covalently bonded model structures as G9–AB–G9 to determine the electronic coupling between contacts, since such coupling is at least one factor controlling charge transport through the junction.

Consider first the G9–AB–G9 model of Figure 1, in which the distance between the nearest carbon atoms in the two G9 rings in the optimized structure is 1.21 nm. Table 3 lists the H, H−1, and H−2 orbital energies and *t* values for the indicated pairs of orbitals. With the AB molecule absent, *t*_H/H−1_ for two G9s with the same spacing in edge orientation from Table 2 is negligible (below 0.1 meV), while with the AB present *t*_H/H−1_ is 3 meV. Recall that the H and H−1 orbitals of G9–AB–G9 are derived from the original HOMO orbitals of G9, and the low *t*_H/H−1_ indicates weak interactions between the two G9 “contacts”. However, as is apparent in Figure 5, the H−2 orbital spans both G9s and the AB molecule, so the coupling between H−2 and H−3 might be more relevant to transport. *t*_H-2/H-3_ is 111 meV, implying significantly stronger coupling compared to the same parameter for unbonded G9s (*t*_H−2/H−3_ < 0.1 meV). Note also that H−2 and H−3 in the G9–AB–G9 system have electron density on both G9 contacts as well as the molecule, which may indicate their likely involvement in transport. Also shown in Table 3 is the effect of rotation of the plane of the AB molecule while the G9 planes are kept parallel. Not surprisingly, *t*_H/H−1_ remains small (below 5 meV) for the full range of planar to orthogonal planes, while *t*_H−2/H−3_ decreases from 187 meV for the planar case to less than 1 meV for dihedral angles of 90°. In our previous theoretical analysis of electronic interactions between the larger G54 graphene fragment and AB, we proposed that the tunneling barrier is related to the offset between the G9–AB HOMO and the G9–AB orbital having significant electron density on the AB molecule [34]. This postulate is consistent both with transport measurements and with independent evaluations by ultraviolet photoelectron spectroscopy and photocurrent measurements [31,51–52]. For the G9–AB–G9 model, this offset would be approximated by the difference between the HOMO and H−2 orbitals, or 0.96 eV for the optimized case. The offset varies with dihedral angle, from 0.83 to 1.25 eV for the planar to the orthogonal structures. Our previous postulate that the orbital determining the tunneling barrier is the closest G9–AB orbital with electron density on the AB moiety can now be enhanced by the further postulate that the tunneling orbital span both the entire G9–AB–G9 system, as shown in Figure 4 for the H−2 orbital. While the LUMO orbitals can also show electronic coupling across the entire junction (apparent in Figure 4), they are energetically less favorable, with an offset from the electrode Fermi level of ca. 2.3 eV in G9–AB–G9 compared to approx. 1 eV for the H−2 orbital.

**Table 3:** Orbital energies for G9–AB–G9.

molecule	gap between C–C, nm	dihedral angle between G9s, °	LUMO, eV	HOMO, eV	H−1, eV	H−2, eV	*t*_H/H−1_, meV	*t*_H−2/H−3_, meV	*t*_L/L+1_, meV

G9–AB–G9 37° dihedral angle (opt)	1.208	15	−2.410	−4.747	−4.753	−5.708	3.0	110.9	38.9

G9–AB–G9 90° dihedral angle	1.208	0	−2.316	−4.733	−4.733	−5.987	0.1	0.8	1.5
G9–AB–G9 75°	1.208	0	−2.321	−4.734	−4.736	−5.948	1.1	20.1	3.1
G9–AB–G9 60°	1.208	0	−2.351	−4.738	−4.742	−5.854	1.9	66.3	15.6
G9–AB–G9 45°	1.208	0	−2.391	−4.744	−4.748	−5.760	2.0	111.2	31.8
G9–AB–G9 37° (opt)	1.208	15	−2.410	−4.747	−4.753	−5.708	3.0	110.9	38.9
G9–AB–G9 20°	1.208	0	−2.451	−4.752	−4.760	−5.627	4.1	170.9	54.7
G9–AB–G9 0° (planar)	1.208	0	−2.468	−4.753	−4.763	−5.586	4.9	187.1	62.0

G9–(AB)_2_–G9 opt	2.268	46	−2.567	−4.759	−4.761	−5.659	0.7	119.0	105.0
G9–(AB)_3_–G9 opt	3.324	12.4	−2.665	−4.764	−4.765	−5.634	0.4	91.6	128.0
G9–(AB)_4_–G9 opt	4.386	13	−2.716	−4.765	−4.766	−5.622	0.3	68.0	103.0
G9–(AB)_5_–G9 opt	5.008	8.6	−2.748	−4.767	−4.767	−5.616	0.1	51.0	78.9
G9–AB–G9 planar	1.208	0	−2.468	−4.753	−4.763	−5.586	4.9	187.1	62.0
G9–(AB)_2_–G9 planar	2.268	0.3	−2.644	−4.769	−4.771	−5.525	1.4	151.2	131.8
G9–(AB)_3_–G9 planar	3.382	0.5	−2.746	−4.774	−4.775	−5.500	0.5	109.9	146.3
G9–(AB)_4_–G9 planar	4.389	1.2	−2.805	−4.776	−4.776	−5.487	0.3	79.6	117.3
G9–(AB)_5_–G9 planar	5.011	0.4	−2.840	−4.779	−4.779	−5.480	<0.1	59.6	90.6

**Figure 6:**
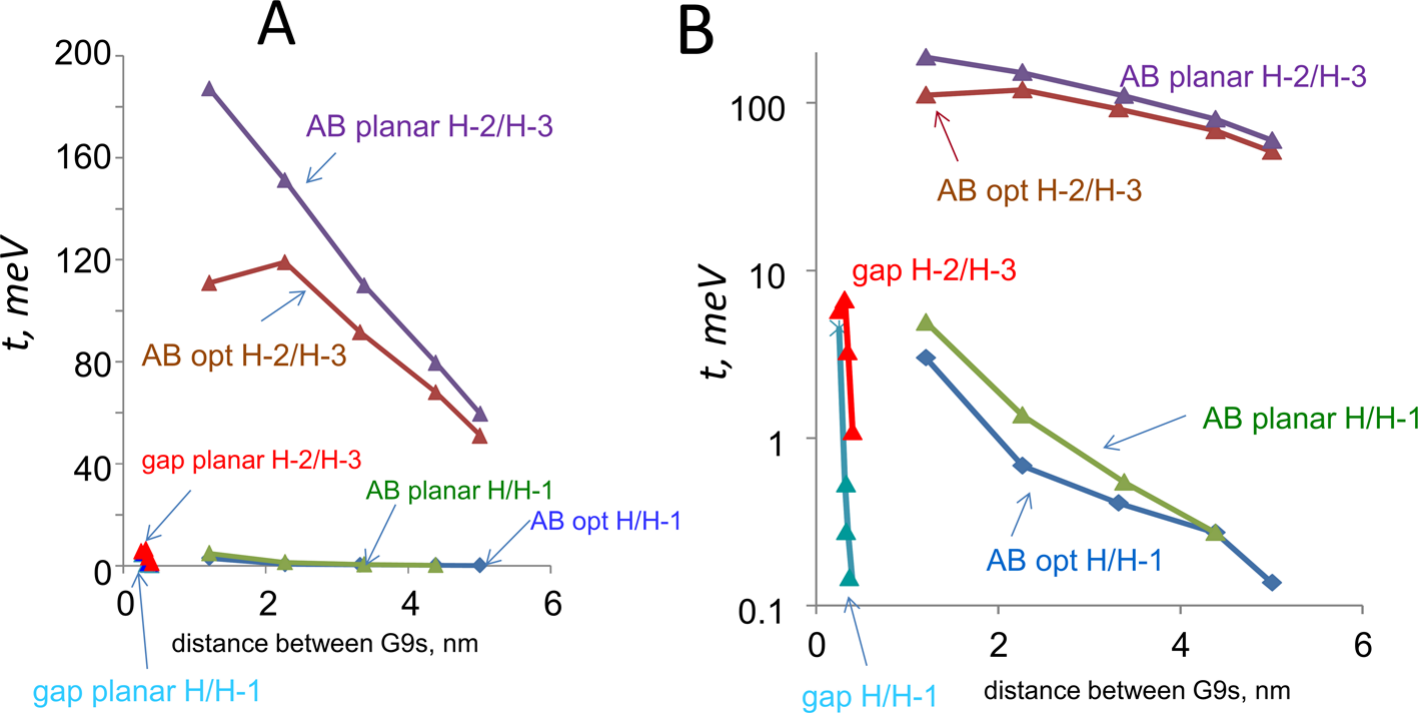
Comparisons of calculated electronic coupling *t* values in meV for G9–(AB)*_n_*–G9 and edge-oriented G9–gap–G9 structures. A) *t* for the indicated orbital combinations for G9–G9 gaps of varying width and for G9–(AB)*_n_*–G9 with varying numbers of AB subunits. *t* was determined between the HOMO and H−1 orbitals for the system containing two G9 units (labeled H/H−1) and for H−2/H−3 orbitals, as indicated. “opt” indicates optimized structures and “planar” structures were forced planar after optimization. B) same data as in panel A, but plotted on a logarithmic Y-axis. X axis is distance between the closest carbon atoms in the G9 fragments.

The model was extended to oligomers of AB between the G9 contacts, in order to assess the effect of molecular layer thickness. Oligomers were assumed to be bonded at the ends of the AB molecules (i.e., para to the azo group) and the optimized structures of G9–(AB)*_n_*–G9 were determined for *n* = 1–5. Selected orbital energies and *t* values appear in Table 3, and more details are included in Table S1 (Supporting Information File 1). The electronic coupling between the G9 molecules decreases as the molecule becomes longer, and the H/H−1 coupling is always much less than the H−2/H−3 coupling. Electronic coupling for the AB oligomers is compared to that for a vacuum gap in Figure 6, with the log axis of Figure 6B showing the very weak coupling for the vacuum gap. Note that the changes in *t*_H−2/H−3_ between the optimized and planar geometries are small compared to the difference between the presence and absence of the AB molecule.

**Figure 7:**
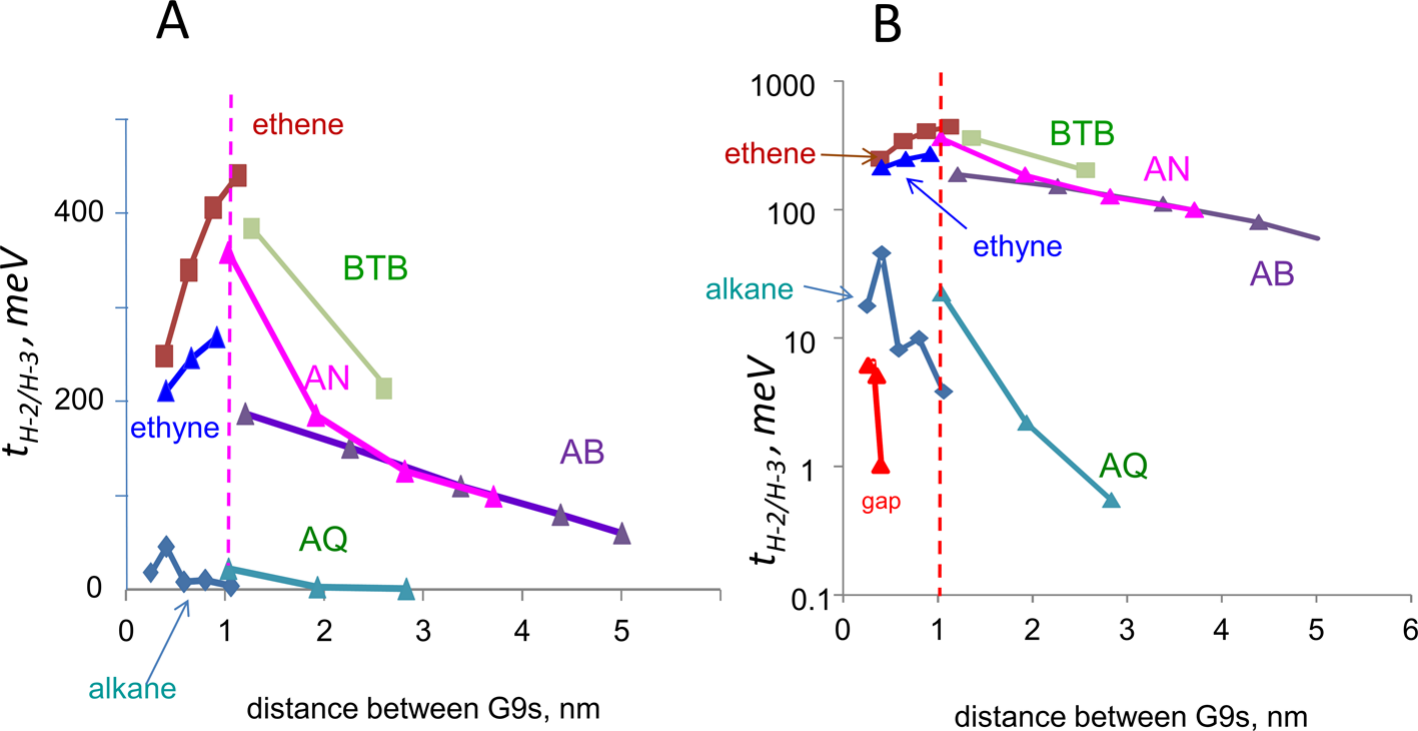
A) *t*_H−2/H−3_ calculated for the planar geometries of the indicated G9–molecule–G9 clusters with various molecular oligomers with lengths from 0.2 to 5 nm. B) same data as panel A on a log scale. BTB = bis(thienyl)benzene, AN = 2-anthracene, AB = azobenzene, ethene = *trans*-oligoethenylenes, ethyne = oligoethynylenes, AQ = anthraquinone.

In order to assess the effects of molecular structure on electronic coupling, several series of oligomers between G9 fragments were calculated, which differ in the degree of conjugation, including alkanes, alkene, and alkynes, with the results summarized in Table 4 and Figure 7. The optimized geometries for G9–molecule–G9 models with variations in molecular structure often have significant dihedral angles between the G9 planes, which complicates interpretation of molecular structural effects on electronic coupling. For this reason, further comparisons considered below were made for the planar configurations of G9–molecule–G9 structures. The real system has a range of dihedral angles, of course, but the effect of dihedral angle on coupling is generally minor compared to changes in conjugation and length. The large Table S2 (Supporting Information File 1) lists orbital energies and coupling calculated for both the optimized and planar configurations for a wide range of model structures. The G9 rings were close to coplanar for both the optimized and planar structures, and some of the alkanes were chosen to be rigid, such as cyclohexane and decalin. For the conjugated systems, the G9–molecule–G9 system was first optimized, then the dihedral angles forced to zero to form a planar structure and the energies recalculated.

**Table 4:** Orbital energies and coupling for planar G9–molecule–G9 systems.

molecule	distance between G9s, nm	dihedral angle between G9s, degrees	HOMO, eV	H−1, eV	H−2, eV	*t*_H/H−1_, meV	*t*_H−2/H−3_, meV	*t*_L/L+1_, meV

alkanes								
G9–CH_2_–G9 planar^a^	0.256	17.5	−4.693	−4.710	−5.901	8.6	17.7	7.9
G9–ethane–G9 planar	0.41	1	−4.673	−4.702	−5.871	14.4	45.6	38.2
G9–cyclohexane–G9 planar^b^	0.59	2	−4.675	−4.678	−5.903	1.9	9.9	5.4
G9–decalin–G9 planar^b^	0.821	1	−4.653	−4.654	−5.889	0.4	1.8	1.2
G9–tri-decalin–G9 planar^b^	1.063	1	−4.648	−4.654	−5.889	3.0	3.8	4.2

(oligo)ethenylenes^c^								
G9–ethene–G9 planar	0.389	0	−4.710	−4.748	−5.520	18.9	248.0	14.7
G9–butadiene–G9 planar	0.635	0	−4.702	−4.739	−5.312	18.5	339.5	13.9
G9–hexatriene–G9 planar	0.882	0	−4.693	−4.735	−5.143	21.1	406.0	17.1
G9–octatetraene–G9 planar	1.13	0	−4.682	−4.731	−5.012	24.8	440.4	22.3

(oligo)ethynylenes								
G9–ethyne–G9 planar	0.407	0	−4.742	−4.773	−5.631	15.6	211.3	19.2
G9–butadiyne–G9 planar	0.664	0	−4.781	−4.807	−5.581	12.9	245.9	19.6
G9–hexatriyne–G9 planar	0.922	0	−4.817	−4.839	−5.551	10.7	268.4	23.8

anthracene^d^								
G9–AN–G9 (corners) planar	1.036	0.2	−4.695	−4.725	−5.199	14.8	358.2	25.7
G9–(AN)_2_–G9 (corners) planar	1.923	0	−4.702	−4.714	−5.071	6.0	185.0	11.0
G9–(AN)_3_–G9 (corners) planar	2.821	0.5	−4.707	−4.727	−5.000	9.7	125.7	16.1
G9–(AN)_4_–G9 (corners) planar	3.713	5.8	−4.706	−4.709	−4.965	1.5	98.8	3.8

anthraquinone^d^								
G9–AQ–G9 planar	1.042	0.46	−4.805	−4.810	−5.955	2.6	22.0	210.2
G9–(AQ)_2_–G9 planar	1.938	2.1	−4.838	−4.839	−6.013	0.1	2.2	99.0
G9–(AQ)_3_–G9 planar	2.836	1.46	−4.851	−4.851	−6.028	0.1	0.5	74.6

bis(thienyl)benzene								
G9–BTB–G9 planar	1.358	1.5	−4.716	−4.741	−5.089	12.5	371.3	10.9
G9–(BTB)_2_–G9 planar	2.565	2.9	−4.718	−4.736	−4.909	8.7	208.2	9.4

^a^Planarity prevented by steric interactions; ^b^G9s parallel, but offset below 0.2 nm; ^c^all in trans-configuration; ^d^AN–AN and AQ–AQ linked at 2-position.

As was the case with AB, similar trends were observed for both the planar and optimized structures, as shown in Table S2 (Supporting Information File 1). Figure 7 compares *t*_H−2/H−3_ for the molecules listed in Table 4 to that for AB and the vacuum gap. In all cases, coupling is stronger with the molecule present, although *t*_H−2/H−3_ covers a wide range from below 1 meV to above 700 meV. With the exceptions of alkenes and alkynes, *t*_H−2/H−3_ decreases as the separation of the G9 “contacts” increases, and logically must reach zero at infinite molecular layer thickness. The increase in coupling with distance calculated for alkenes and alkynes is unexpected, although it is small compared to the differences between alkanes, vacuum, and aromatics. The alkene and alkyne cases are not readily accessible with carbon contacts, hence this unexpected result is difficult to test experimentally. As suggested by a reviewer, it may be an indication that the electronic coupling represented by *t*_H−2/H−3_ is not due simply to coupling between the G9 contacts (which should certainly decrease with distance), but might instead indicate coupling between molecule and G9 orbitals. This possibility is testable by further experiments with variations in molecular structure. For the readily fabricated aromatic molecular junctions, it is likely that the particular orbitals most involved in transport will be revealed by further correlations of calculated orbital energy splitting with experimental results.

The conjugated systems show similar electronic coupling, with a weaker decrease with thickness than the alkanes. The trends in electronic coupling evident in Figure 7B have a striking similarity to experimental results from a variety of laboratories and paradigms [1,5,31,53]. Electron transport across thin organic layers often decays exponentially with layer thickness, with an “attenuation length (β)" equal to the slope of a plot of the natural log of transport rate vs layer thickness. Real alkane junctions exhibit a significantly higher β (8–9 nm^−1^) than conjugated systems (β = 2–4 nm^−1^), and many of the latter have similar β values despite structural differences. AQ is an interesting case, because is it “cross conjugated” with a much smaller *t*_H−2/H−3_ than that for the structurally related anthracene case [54]. Several experimental reports have shown a quite distinct behavior of AQ compared to AN “bridges”, attributed to cross conjugation and quantum interference [54–58]. Since the relationship between *t*_H−2/H−3_ and the attenuation coefficient β depends on the transport mechanism, the similarity between Figure 7B and various experimental β plots should be considered qualitative. Although the model structure is significantly simplified compared to the real carbon/oligomer/carbon MJ, *t*_H−2/H−3_ determined for G9–molecule–G9 structures is at least a guide toward predicting transport in proposed junction structures, or for systems which are difficult to realize experimentally.

### 3 Energy barriers and partial charge transfer

**Figure 8:**
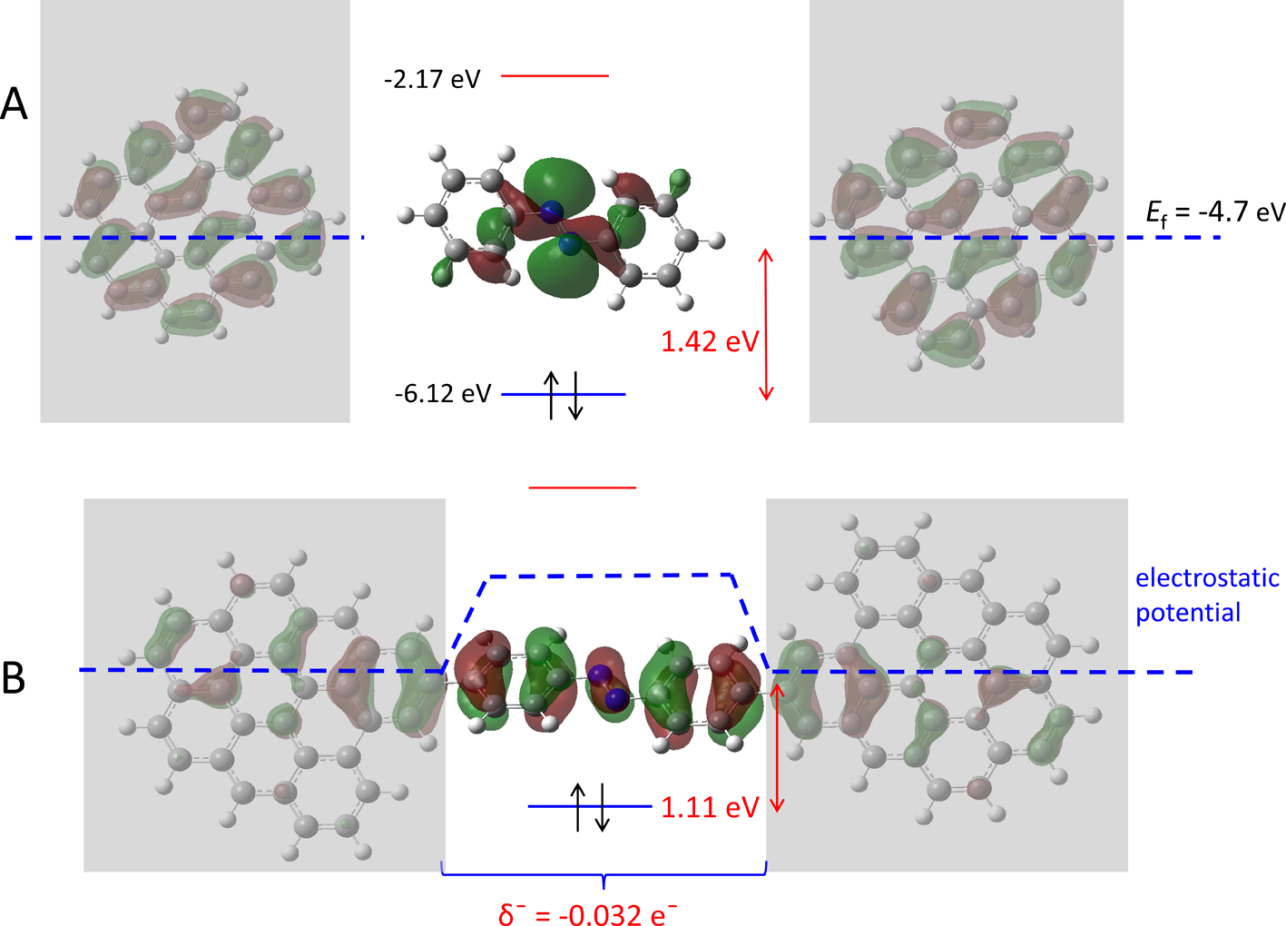
A) Isolated AB and G9 molecules showing the calculated HOMO and LUMO energies relative to the experimentally observed carbon Fermi level of −4.7 eV. Also shown is the 1.42 eV tunneling barrier predicted from the free molecules. B. Schematic of electrostatic potential resulting from charge transfer of 0.032 e^−^ to the AB molecule from the G9s accompanying covalent bond formation. The expected tunneling barrier is reduced to 1.11 eV, as shown.

As noted in the introduction, we have examined the postulate that the energy barrier for tunneling across carbon-based molecular junctions correlates with the difference in energy between the system HOMO and the orbital closest in energy that has significant electron density on the molecule [34]. The arguments of the previous section indicate that electronic coupling is strongest for orbitals that bridge across the entire G9–molecule–G9 structure, often H−2 and H−3 in the structures considered here. It is tempting to conclude that *E*_HOMO_ – *E*_H−2_ represents the magnitude of the tunneling barrier, in which case prediction of the barrier is straightforward for the model system. However, several authors have pointed out that electronic coupling between the contacts and molecules at organic/conductor interfaces can significantly perturb the simple picture, due to local electrostatic effects [36–38,40,59–60]. “Vacuum level alignment” effects on interfacial barriers are often attributed to surface dipoles which cause charge transfer across an interface, resulting in changes in local electrostatic potential [14,38,40]. For the case of G9–AB, if electrons are transferred from G9 to AB when the G9–AB bond is formed, the electrostatic potential of AB increases, thus raising its HOMO level compared to that of the free molecule. Figure 8 shows the effect schematically, starting with two separated G9 planes and an AB molecule with its HOMO at −6.16 eV for the free molecule. The −4.7 eV work function observed experimentally is close to the −4.68 eV HOMO calculated for G9, and both G9s have identical HOMO levels. When the G9–AB–G9 system is formed, DFT predicts that 0.032 e^−^ are transferred to the AB molecules, as shown in Figure 8B.

The local electrostatic potential associated with these electrons shifts all of the orbitals in AB to higher energy, and decreases the transport barrier predicted from the work function and HOMO energy of the separated contact and molecule. Although there is some concern about the accuracy of DFT for predicting Mulliken charges [41], trends are generally reliable, such as the dependence on dihedral angle shown in Figure 3. At least in principle, the DFT-determined energies for G9–molecule–G9 model structures should include such electrostatic effects, including local dipoles and Mulliken charges. As an indication of the magnitude of the effect, Guerrero et al. have provided an expression for predicting the vacuum level shift, Δ, from the charge transferred, *Q*, the dielectric constant, ε, and an estimated interface thickness, δ: Δ = δ*Q*/ε [61]. Using this approach, we predict a vacuum level shift of 0.35 V for ε = 4 and δ = 1 nm, implying that *E*_f_ − *E*_HOMO_ would decrease from the value predicted for the free molecule by 0.35 V. The assumptions required for this calculation do not permit a rigorous quantitative analysis of the effect, but it does indicate that transport barriers can be significantly perturbed by partial charge transfer. We showed previously that the effect is sufficiently large in carbon MJs with aromatic molecular layers that differences in free molecule HOMO levels caused a small effect on junction conductance. Since low HOMO levels cause larger charge transfer than higher HOMO levels, the effect “levels” a range of barriers of 0.7 to 3.0 eV based on the free molecule HOMOs to 1.3 ± 0.2 eV determined experimentally [31].

## Conclusion

The author agrees with the often-quoted words attributed to I. M. Koltoff: “theory guides, experiment decides”. The purpose of the current paper is to identify some of the “guides” that apply to how molecular structure affects electron transport in carbon-based molecular electronic devices. With that objective in mind, several observations are available from the DFT results. First, electronic coupling is an important factor in understanding transport, both coupling between the molecule and the contacts, and between subunits within the molecular layer. Strong coupling indicated by a large *t* value should promote electron transfer for both tunneling and activated “hopping” mechanisms, and decreases with increasing distance between the coupled sites. All molecules examined increase electronic coupling between graphene “contacts” compared to a vacuum gap, but the increase is strongly structure-dependent, as shown in Figure 7B. Although electronic coupling depends on the dihedral angles between aromatic subunits, real experimental systems in use currently have a range of dihedral angles, and device behavior represents an average over this range. Second, the electronic coupling across G9–molecule–G9 is weak for the HOMO and H−1 orbitals, which were the original HOMOs of the isolated G9 fragments. Coupling is much stronger for hybrid orbitals H−2 and H−3, which exhibit electron density on both the G9 and molecule moieties, implying they are more likely involved in transport. For many cases, these orbitals are approximately 1 eV below the system HOMO, and likely represent the orbitals responsible for the experimentally observed barriers of 1.3 ± 0.2 eV [31]. Third, electronic coupling can also result in partial charge transfer between contacts and the molecular layer, which leads to the electrostatic perturbation of the tunneling barrier illustrated in Figure 8. Unfortunately, this effect decreases the influence of electron donating and withdrawing groups on the observed barrier, thus frustrating some attempts to modulate transport with structure. Although the magnitude of the effect is difficult to predict theoretically, it can be measured with various probes such as UPS and inverse photoemission for molecular layers on surfaces [14,31,40,62–63], and photocurrents for intact molecular junctions [51–52]. Finally, the conjugated covalent bond between the aromatic π-systems of the carbon substrate and aromatic molecular layer is responsible for both the strong electronic coupling and the excellent stability of carbon-based molecular junctions. The guidance provided by DFT illustrated here should prove useful for designing new MJ structures which exploit these properties.

## Supporting Information

Supporting Information features the orientations of G9 fragments used to calculate the energies of Table 2 as well as a complete table of orbital energies for the molecules shown in Figure 6 and Figure 7 for both the optimized and planar geometries.

File 1 Additional computational data.

## References

[1] Metzger R M (2015). Chem Rev.

[2] McCreery R L, Yan H, Bergren A J (2013). Phys Chem Chem Phys.

[3] McCreery R L, Bergren A J (2009). Adv Mater.

[4] Heath J R (2009). Annu Rev Mater Res.

[5] Amdursky N, Marchak D, Sepunaru L, Pecht I, Sheves M, Cahen D (2014). Adv Mater.

[6] Aradhya S V, Meisner J S, Krikorian M, Ahn S, Parameswaran R, Steigerwald M L, Nuckolls C, Venkataraman L (2012). Nano Lett.

[7] Chen W, Widawsky J R, Vázquez H, Schneebeli S T, Hybertsen M S, Breslow R, Venkataraman L (2011). J Am Chem Soc.

[8] Venkataraman L, Park Y S, Whalley A C, Nuckolls C, Hybertsen M S, Steigerwald M L (2007). Nano Lett.

[9] Li Z, Smeu M, Ratner M A, Borguet E (2013). J Phys Chem C.

[10] DiBenedetto S A, Facchetti A, Ratner M A, Marks T J (2009). J Am Chem Soc.

[11] Solomon G C, Andrews D Q, Van Duyne R P, Ratner M A (2008). J Am Chem Soc.

[12] Lindsay S M, Ratner M A (2007). Adv Mater.

[13] Luo L, Choi S H, Frisbie C D (2011). Chem Mater.

[14] Kim B, Choi S H, Zhu X-Y, Frisbie C D (2011). J Am Chem Soc.

[15] Choi S H, Kim B, Frisbie C D (2008). Science.

[16] Yoon H J, Shapiro N D, Park K M, Thuo M M, Soh S, Whitesides G M (2012). Angew Chem, Int Ed.

[17] Cademartiri L, Thuo M M, Nijhuis C A, Reus W F, Tricard S, Barber J R, Sodhi R N S, Brodersen P, Kim C, Chiechi R C (2012). J Phys Chem C.

[18] Nijhuis C A, Reus W F, Whitesides G M (2010). J Am Chem Soc.

[19] Akkerman H B, Kronemeijer A J, Harkema J, van Hal P A, Smits E C P, de Leeuw D M, Blom P W M (2010). Org Electron.

[20] Van Hal P A, Smits E C P, Geuns T C T, Akkerman H B, De Brito B C, Perissinotto S, Lanzani G, Kronemeijer A J, Geskin V, Cornil J (2008). Nat Nanotechnol.

[21] Akkerman H B, Blom P W M, de Leeuw D M, de Boer B (2006). Nature.

[22] Green J E, Wook Choi J, Boukai A, Bunimovich Y, Johnston-Halperin E, DeIonno E, Luo Y, Sheriff B A, Xu K, Shik Shin Y (2007). Nature.

[23] DeIonno E, Tseng H-R, Harvey D D, Stoddart J F, Heath J R (2006). J Phys Chem B.

[24] Vilan A, Yaffe O, Biller A, Salomon A, Kahn A, Cahen D (2010). Adv Mater.

[25] Yu X, Lovrinčić R, Kraynis O, Man G, Ely T, Zohar A, Toledano T, Cahen D, Vilan A (2014). Small.

[26] Li Y, Calder S, Yaffe O, Cahen D, Haick H, Kronik L, Zuilhof H (2012). Langmuir.

[27] Seitz O, Vilan A, Cohen H, Hwang J, Haeming M, Schoell A, Umbach E, Kahn A, Cahen D (2008). Adv Funct Mater.

[28] McCreery R, Mirkin M V, Amemiya S (2015). Electron Transport and Redox Reactions in Solid-State Molecular Electronic Devices. Nanoelectrochemistry.

[29] McCreery R, Bergren A, Morteza-Najarian A, Sayed S Y, Yan H (2014). Faraday Discuss.

[30] Yan H, Bergren A J, McCreery R, Della Rocca M L, Martin P, Lafarge P, Lacroix J C (2013). Proc Natl Acad Sci U S A.

[31] Sayed S Y, Fereiro J A, Yan H, McCreery R L, Bergren A J (2012). Proc Natl Acad Sci U S A.

[32] Bergren A J, McCreery R L (2011). Annu Rev Anal Chem.

[33] Anariba F, Steach J K, McCreery R L (2005). J Phys Chem B.

[34] Kondratenko M, Stoyanov S R, Gusarov S, Kovalenko A, McCreery R L (2015). J Phys Chem C.

[35] Bergren A J, McCreery R L, Stoyanov S R, Gusarov S, Kovalenko A (2010). J Phys Chem C.

[36] Salomon A, Boecking T, Seitz O, Markus T, Amy F, Chan C, Zhao W, Cahen D, Kahn A (2007). Adv Mater.

[37] Cahen D, Kahn A (2003). Adv Mater.

[38] Braun S, Salaneck W R, Fahlman M (2009). Adv Mater.

[39] Ishii H, Sugiyama K, Ito E, Seki K (1999). Adv Mater.

[40] Hwang J, Wan A, Kahn A (2009). Mater Sci Eng, R.

[41] Saha S, Roy R K, Ayers P W (2009). Int J Quantum Chem.

[42] McCreery R L, Bergren A J, Chehimi M M (2012). Diazonium reagents in molecular electronics. Aryl Diazonium Salts: New Coupling Agents in Polymer and Surface Science.

[43] Yan H, Bergren A J, McCreery R L (2011). J Am Chem Soc.

[44] Wu J, McCreery R L (2009). J Electrochem Soc.

[45] Wu J, Mobley K, McCreery R L (2007). J Chem Phys.

[46] McCreery R L, Wu J, Kalakodimi R P (2006). Phys Chem Chem Phys.

[47] Kumar R, Yan H, McCreery R L, Bergren A J (2011). Phys Chem Chem Phys.

[48] Mujica V, Ratner M A (2001). Chem Phys.

[49] Lacroix J C, Chane-Ching K I, Maquère F, Maurel F (2006). J Am Chem Soc.

[50] Sancho-García J C, Pérez-Jiménez A J (2008). J Chem Phys.

[51] Fereiro J A, Kondratenko M, Bergren A J, McCreery R L (2015). J Am Chem Soc.

[52] Fereiro J A, McCreery R L, Bergren A J (2013). J Am Chem Soc.

[53] Bonifas A P, McCreery R L (2010). Nat Nanotechnol.

[54] Fracasso D, Valkenier H, Hummelen J C, Solomon G C, Chiechi R C (2011). J Am Chem Soc.

[55] Guédon C M, Valkenier H, Markussen T, Thygesen K S, Hummelen J C, van der Molen S J (2012). Nat Nanotechnol.

[56] Rabache V, Chaste J, Petit P, Della Rocca M L, Martin P, Lacroix J-C, McCreery R L, Lafarge P (2013). J Am Chem Soc.

[57] Valkenier H, Guédon C M, Markussen T, Thygesen K S, van der Molen S J, Hummelen J C (2014). Phys Chem Chem Phys.

[58] Darwish N, Díez-Pérez I, Da Silva P, Tao N, Gooding J J, Paddon-Row M N (2012). Angew Chem, Int Ed.

[59] Van Dyck C, Ratner M A (2015). Nano Lett.

[60] Van Dyck C, Geskin V, Cornil J (2014). Adv Funct Mater.

[61] Guerrero A, Marchesi L F, Boix P P, Ruiz-Raga S, Ripolles-Sanchis T, Garcia-Belmonte G, Bisquert J (2012). ACS Nano.

[62] Shpaisman H, Seitz O, Yaffe O, Roodenko K, Scheres L, Zuilhof H, Chabal Y J, Sueyoshi T, Kera S, Ueno N (2012). Chem Sci.

[63] Segev L, Salomon A, Natan A, Cahen D, Kronik L, Amy F, Chan C K, Kahn A (2006). Phys Rev B: Condens Matter Mater Phys.

