# Supplementary material for: Effects of electronic coupling and electrostatic potential on charge transport in carbon-based molecular electronic junctions
Source: Beilstein J Nanotechnol. 2016 Jan 11;7:32–46. doi: 10.3762/bjnano.7.4 (PMC4734416; doi:10.3762/bjnano.7.4)
Supplement: File 1 — Additional computational data. [file Beilstein_J_Nanotechnol-07-32-s001.pdf]

Supporting Information

for

Effects of electronic coupling and electrostatic potential on charge transport in carbon-based molecular electronic junctions

Richard L. McCreery*

Address: Department of Chemistry, University of Alberta, Edmonton, AB, Canada and National Institute for Nanotechnology, National Research Council Canada

Email: Richard L. McCreery* - richard.mccreery@ualberta.ca

* Corresponding author

Additional computational data

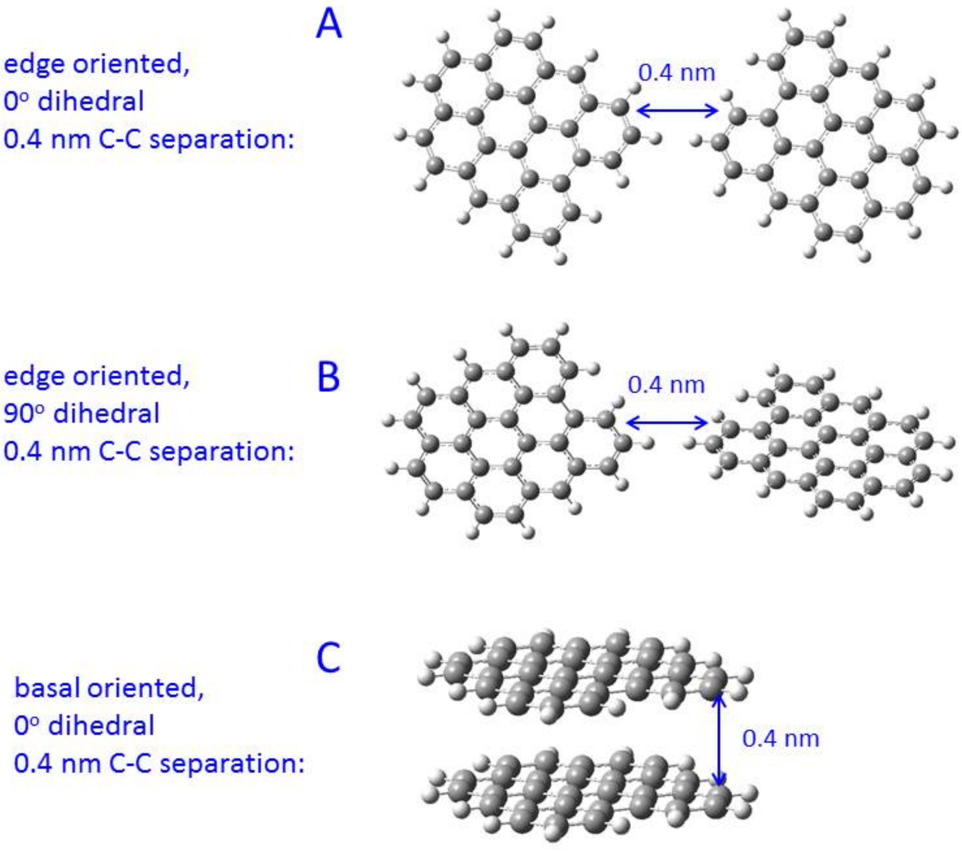

Figure S1: Orientations of G9 molecules used to calculate the results in Table 2 of the main text.

Table S1: Orbital energies and coupling for G9–molecule–G9 systems in optimized and planar configurations.

molecule	distance between G9s, nm	dihedral between G9s, degrees	L+1	LUMO	HOMO	H-1	H-2	H-3	$t_{H/H-1}$	$t_{H-2/H-3}$	$t_{L/L+1}$
			(eV)	(eV)	(eV)	(eV)	(eV)	(eV)	(meV)	(meV)	(meV)
**alkanes**											
G9-CH2-G9 opt	0.256	93	-2.214	-2.291	-4.673	-4.702	-5.871	-5.962	14.4	45.6	38.2
G9- ethane-G9 opt incompl.	0.41	17	-2.255	-2.271	-4.543	-4.550	-5.968	-5.984	3.7	8.0	7.9
G9-cyclohexane-G9 opt	0.59	6	-2.247	-2.257	-4.675	-4.678	-5.903	-5.923	1.9	9.9	5.3
G9-decalin-G9 opt	0.803	14	-2.237	-2.246	-4.657	-4.662	-5.897	-5.907	2.6	5.0	4.6
G9-tri-decalin-G9 opt	1.063	1	-2.226	-2.228	-4.640	-4.648	-5.887	-5.890	4.2	1.5	1.1
G9-CH2-G9 planar[a]	0.256	18	-2.241	-2.256	-4.693	-4.710	-5.901	-5.936	8.6	17.7	7.9
G9- ethane-G9 planar	0.41	1	-2.214	-2.291	-4.673	-4.702	-5.871	-5.962	14.4	45.6	38.2
G9-cyclohexane-G9 planar[b]	0.59	2	-2.247	-2.257	-4.675	-4.678	-5.903	-5.923	1.9	9.9	5.4
G9-decalin-G9 planar[b]	0.821	1	-2.227	-2.230	-4.653	-4.654	-5.889	-5.893	0.4	1.8	1.2
G9-tri-decalin-G9 planar[b]	1.063	1	-2.229	-2.237	-4.648	-4.654	-5.889	-5.896	3.0	3.8	4.2
**oligo(ethenylenes)**[c]											
G9-ethylene-G9	0.389	23	-2.284	-2.310	-4.707	-4.745	-5.535	-6.015	19.0	239.6	13.3
G9- butadiene-G9 opt	0.635	18	-2.284	-2.311	-4.702	-4.738	-5.321	-6.016	18.0	347.5	13.7

G9- hexatriene-G9 opt	0.882	15	-2.282	-2.317	-4.693	-4.735	-5.147	-5.955	20.5	404.2	17.1
G9- octatetraene-G9 opt	1.13	10	-2.283	-2.327	-4.681	-4.731	-5.013	-5.893	24.8	440.0	22.2
G9- ethene-G9 planar	0.389	0	-2.282	-2.312	-4.710	-4.748	-5.520	-6.016	18.9	248.0	14.7
G9- butadiene-G9 planar	0.635	0	-2.283	-2.311	-4.702	-4.739	-5.312	-5.991	18.5	339.5	13.9
G9-hexatriene-G9 planar	0.882	0	-2.282	-2.317	-4.693	-4.735	-5.143	-5.955	21.1	406.0	17.1
G9- octatetraene-G9 planar	1.13	0	-2.283	-2.327	-4.682	-4.731	-5.012	-5.893	24.8	440.4	22.3
**oligo(ethynylenes)**											
G9- ethyne-G9 opt	0.406	11	-2.322	-2.359	-4.742	-4.773	-5.635	-6.052	15.4	208.4	18.8
G9- butadiyne-G9 opt	0.664	12	-2.363	-2.401	-4.781	-4.806	-5.585	-6.071	12.5	242.6	19.2
G9- hexatriyne-G9 opt	0.922	28	-2.402	-2.443	-4.819	-4.837	-5.572	-6.034	9.4	231.3	20.8
G9- ethyne-G9 planar	0.407	0	-2.322	-2.360	-4.742	-4.773	-5.631	-6.053	15.6	211.3	19.2
G9- butadiyne-G9 planar	0.664	0	-2.362	-2.402	-4.781	-4.807	-5.581	-6.073	12.9	245.9	19.6
G9-hexatriyne-G9 planar	0.922	0	-2.400	-2.447	-4.817	-4.839	-5.551	-6.088	10.7	268.4	23.8
**anthracene**[d]											
G9-AN-G9 (corners) opt	1.036	78	-2.299	-2.315	-4.709	-4.720	-5.234	-5.942	5.3	354.0	8.0
G9-2AN-G9 (corners) opt	1.923	112	-2.303	-2.311	-4.711	-4.715	-5.106	-5.391	2.0	142.9	3.7
G9-3AN-G9 (corners) opt	2.798	32	-2.305	-2.308	-4.712	-4.714	-5.045	-5.266	0.8	110.8	1.8
G9-4AN-G9 (corners) opt	3.662	22	-2.304	-2.306	-4.713	-4.714	-5.014	-5.181	0.4	83.8	1.1
G9-AN-G9 (corners) planar	1.036	0	-2.311	-2.363	-4.695	-4.725	-5.199	-5.915	14.8	358.2	25.7
G9-2AN-G9 (corners) planar	1.923	0	-2.328	-2.350	-4.702	-4.714	-5.071	-5.441	6.0	185.0	11.0

G9-3AN-G9 (corners) planar	2.821	1	-2.308	-2.340	-4.707	-4.727	-5.000	-5.251	9.7	125.7	16.1
G9-4AN-G9 (corners) planar	3.713	6	-2.336	-2.344	-4.706	-4.709	-4.965	-5.163	1.5	98.8	3.8
**anthraquinone**[d]											
G9-AQ-G9 opt	1.042	74	-2.388	-2.791	-4.800	-4.801	-5.998	-6.022	0.8	11.7	201.6
G9-2AQ-G9 opt	1.933	96	-2.808	-2.997	-4.828	-4.829	-6.040	-6.042	0.5	1.0	94.6
G9-3AQ-G9 opt	2.854	86	-2.954	-3.109	-4.838	-4.840	-6.052	-6.052	0.8	0.1	78.0
G9-AQ-G9 planar	1.042	0	-2.397	-2.817	-4.805	-4.810	-5.955	-5.999	2.6	22.0	210.2
G9-2AQ-G9 planar	1.938	2	-2.832	-3.030	-4.838	-4.839	-6.013	-6.017	0.1	2.2	99.0
G9-3AQ-G9 planar	2.836	1	-2.995	-3.144	-4.851	-4.851	-6.028	-6.030	0.1	0.5	74.6
**bis(thienyl)benzene**											
G9-BTB-G9 opt	1.354	11	-2.312	-2.342	-4.721	-4.744	-5.175	-5.891	11.6	357.8	15.1
G9-2BTB-G9 opt	2.539	11	-2.319	-2.349	-4.727	-4.743	-4.949	-5.379	7.9	215.0	14.8
G9-BTB-G9 planar	1.358	2	-2.322	-2.344	-4.716	-4.741	-5.089	-5.831	12.5	371.3	10.9
G9-2BTB-G9 planar	2.565	3	-2.330	-2.349	-4.718	-4.736	-4.909	-5.326	8.7	208.2	9.4

[a]planarity prevented by steric interactions; [b]G9s parallel, but offset <0.2 nm; [c]all in *trans*-configuration; [d]AN–AN and AQ–AQ linkages at 2 position in all cases
